# Hemophagocytic lymphohistiocytosis and myelodysplastic syndrome: a case report and review of the literature

**DOI:** 10.1186/s13256-020-02623-2

**Published:** 2021-03-01

**Authors:** Y. Sun, C. Blieden, B. Y. Merritt, R. Sosa, Gustavo Rivero

**Affiliations:** 1 Section of Hematology/Oncology, Baylor St Luke Medical Center, Houston, TX 77030 USA; 2 Department of Molecular and Human Genetics, Baylor Genetics, Baylor College of Medicine, Houston, TX USA; 3 Department of Pathology and Immunology, Baylor St. Luke’s Medical Center, Baylor College of Medicine, Houston, TX USA; 4 The Dan L. Duncan Comprehensive Cancer Center at Baylor College of Medicine, 1 Baylor Plaza, Houston, TX 77030 USA

**Keywords:** Hemophagocytic lymphohistiocytosis, Myelodysplastic syndrome, Hyperinflammation

## Abstract

**Background:**

Hemophagocytic lymphohistiocytosis (HLH) is characterized by hyperinflammation and life-threatening cytopenias. Survival is poor, and management is pivotal on rapid identification of the disease. HLH is associated with hematologic malignancies, however correlation with myelodysplastic syndromes (MDS) is exceedingly unusual. Although minimizing overwhelming hyperinflammation by treating hemophagocytosis are central for HLH outcome, there is urgent necessity to identify potential initiating mechanisms that could assist in therapy design.

**Case description:**

Here, we describe an elderly African American patient who developed rapid onset of cytopenias and coagulopathy associated with hepatic and bone marrow hemophagocytosis. We analyze four additional similar cases to isolate clinical, laboratory and cytogenetic findings expected in patients exhibiting concurrent HLH and MDS. HLH linked with MDS retains common HLH features associated with systemic hyperinflammation such as fever, hypotension, hepatosplenomegaly, hyperferritinemia, coagulopathy and rapidly evolving cytopenias. Typical MDS chromosomic abnormality such as trisomy 8 was frequently observed in our studied cases.

**Conclusion:**

Our case describes difficulties while managing HLH in MDS patients. Diagnosis should be based on identifying HLH appropriate criteria and if possible karyotypic abnormalities normally observed in MDS.

## Introduction

Hemophagocytic lymphohistiocytosis (HLH) is characterized by deregulated immunity and end organ damage. Primary HLH is linked with autosomal recessive and X-linked mutations. Secondary HLH results from predisposing conditions. Both primary and secondary HLH may be activated by an infections and malignancies. [1] The association of HLH with hematologic malignancies is accepted. However, HLH initiated by myelodysplastic syndrome (MDS) is exceedingly unusual. Although dysplasia is observed in patients with hemophagocytosis, a hyperinflammatory entity characterized by dysplastic cytopenias, MDS like karyotypic abnormality and hemophagocytosis suggests a different spectrum of disease, in which HLH originates from a clonal disorder. In this report, we present an elderly female who fulfilled HLH 2004 criteria exhibiting erythroid and megakaryocytic dysplasia associated with trisomy 8 [+ 8]. Additionally, we examine published cases in English literature to identify clinical, laboratory and cytogenetic features observed in MDS patients fulfilling HLH 2004 criteria.

## Case presentation

**Figure 1.**
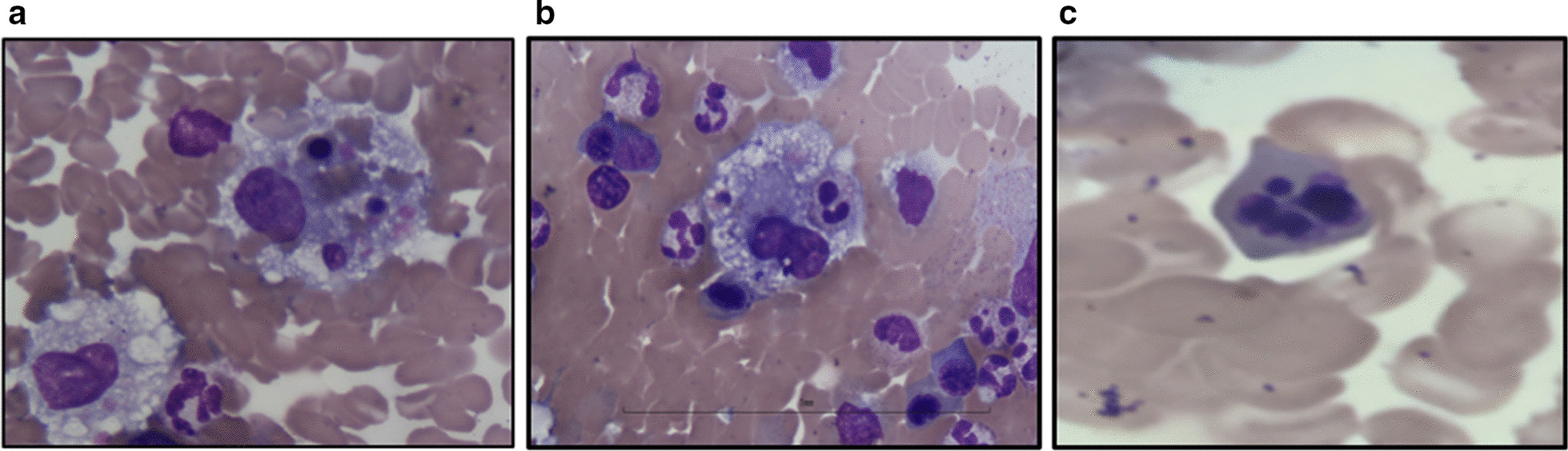
Bone marrow hemophagocytosis and dysplastic changes in a trisomy 8 myelodysplastic syndrome patient. **a** Bone marrow aspirate smear revealing active hemophagocytosis. Erythroid cells are phagocytized by histiocytic cells. **b** Bone marrow aspirate showing neutrophil phagocytized by histiocytic cell. Peripherally to hemophagocytosis, erythroid precursor shows megaloblastic changes. Additionally, hyposegmented neutrophils are observed. **c** Nucleated red cell showing nuclear fragmentation with dysmorphic features suggesting dysplasia

An 83-year-old African American female presented with lethargy, temperature of 102.9° F and tachycardia. After 9 days of broad-spectrum antibiotics, she developed hemodynamic instability requiring vasopressors, worsening liver function (peak bilirubin of 12.5 mg/dL, Aspartate aminotransferase (AST) of 689 IU/L, Alanine aminotransferase (ALT) of 239 IU/L) and leukocytosis of 16,000/uL. Her hemoglobin was 8.9/uL and platelets had fallen to 26,000/uL. Disseminated intravascular coagulation (DIC) was considered given progressive severe thrombocytopenia of 13,000 U/L, fibrinogen < 70 mg/dL and d-dimer of 12.4 mg/L. Her ferritin was 9479 ng/mL and fasting triglycerides (TAG) were 321 mg/dL. Soluble interleukin-2Rα (CD25) was < 38 pg/dL. Her human immunodeficiency virus (HIV), rapid influenza A/B, hepatitis B/C serologies were all negative. Epstein-barr virus (EBV) viral load was negative. Given concern autoimmune hepatitis, a liver biopsy showed Kupffer cell hypertrophy with hemophagocytosis. Bone marrow biopsy demonstrated hemophagocytosis (Fig 1a, b). In addition, significant erythroid nuclear fragmentation and karyorrhexis were observed (Fig 1c). Marrow cytogenetics showed 47, XX +8 [6], 46, XX [5]. Next generation sequencing (NGS) including *CSFR1*, *SF3B1, SRSF2, U2AF1, NRAS, KRAS, FLT3, JAK2, KIT, PHF6, PDGFRA, CDKN2A, IDH1, IDH2, TET2, EZH2, CEBPA, EP300, PTPN11, P53, CREBBP, IKZF1, IKZF3, NOTCH1, RUNX1, WT1 and NPM1* showed *DNMT3A* p.Arg736His (c.2207G>A) and *DNMT3A* p.Leu859Ter (c.2576T>A) at allele frequencies of 2.7 and 2.4 %, respectively. In view of her hemophagocytosis, cytopenias, high temperature, abnormal liver function test, low fibrinogen and elevated fasting triglycerides and ferritin, she fulfilled 5/8 HLH 2004 criteria. HLH-94 regimen was initiated with dexamethasone and etoposide. She developed neutropenic sepsis and etoposide was stopped. Blood cultures were positive for Escherichia coli. Patient expired after developing hemodynamic instability.

## Methods

In addition to our case, three additional previously published reports were included in our reviewed cases. Clinical, laboratory and karyotypic data was analyzed to investigate potential features commonly observed in patients presenting with hyperinflammation and MDS. Data aggregation from four cases presenting with HLH and MDS allowed identification of clinical outcome in patients receiving HLH directed therapy or alternative regimens.

### Cohort analysis

HLH is a deadly syndrome. If untreated, survival is less than 2 months [2]. Here, we describe an elderly female exhibiting hyperinflammation, refractory cytopenias, erythroid dysplasia, expansion of + 8 metaphases and bone marrow/hepatic hemophagocytosis suggesting HLH associated with MDS (HLH-MDS). In addition to our patient, three previously published HLH cases associated with MDS were included in study cohort (Table 1). 7/8 HLH criteria were observed in two (50%), and one (25%) case with 6/8 and 5/8 criteria, each. Our report and published cases are novel since 3 of 4 (75%) patients harbored a karyotypic abnormality highlighting the clonal nature of the disease. A detectable chromosomic abnormality suggests that HLH was systemically initiated by MDS rather than representing a reactive phenomenon [3-5]. In reviewed cases, patients were older than 60 years with exception of one pediatric patient. International prognostic score system (IPSS) was intermediate-1 and high-risk (2 cases, each). + 8 was detected as sole abnormality or within a complex metaphase in 75% of cases. Most of the patients succumbed to the disease, except one case treated with high dose methylprednisolone, cyclosporine (CSA) decitabine. Tamamyan *et al.* described 33 cases of HLH, of which 3(9%) exhibited concurrent MDS, although only one was identified by HLH 2004 [6]. Karyotypic abnormalities were not reported. As in our case, the author reported that HLH linked with MDS represented a fatal condition.

**Table 1:** Myelodysplastic syndrome cases associated with hemophagocytic Lymphohistiocytosis (HLH)

	Age (years)	Fever	ANC (/mL)	Hb (g/dL)	Platelet (K/UL)	Ferritin (ng/dL)	Fibrinogen (mg/dL)	TAG (mg/dL)	IL-2 (pg/dL)	Hemophagocytosis	Marrow blast (%)	Karyotype	R-IPSS	Hepatomegaly/splenomegaly	HLH-2004 (points)	Outcome
1	8	Yes	400	10.6	49	1070	256	306	4250	Yes	2.2	46, XX, + 8 [20]	Inter-mediate	Yes/yes	5	Alive
2	60	Yes	6800	6.5	14	649	NA	NA	4054	Yes	2	Complex [including + 8]^a^	Very high	Yes/yes	5	Alive
3	68	NA	NA	10.5	45	24,316	129	NA	1025	Yes	0	46, XY	Low	No/yes	5	Died
Case	83	Yes	8900	8.9	13	9479	70	321	34	Yes	0	47, XX, + 8 [6], 46, XX [5]	Low	No/no	6	Died

*ANC* absolute neutrophil count, *Hb* hemoglobin, *TAG* triglycerides, *IL2* Interleukin 2 soluble receptor, *IPSS* International Prognostic Score System, *NA* not available

^a^Patient karyotype = 54-57,XY + 1 [2], + 3 [2], + [4], + 8 [4].add (9) (p22) [2], + 11[3].add (15) (p.11.2) [4], add (16) (q24) [4], add (19) (p13.1) [4], add (20) (p13) [4],?21 [2],2–5mar[cp4]/46,XY [8]

## Conclusions

Efforts to elucidate pathogenesis of HLH demonstrate that expansion of CD8^+^ cytotoxic T cells, low Treg frequencies and cytokine storm are frequently observed [7]. In MDS, similar clonal T cell expansion and decreased Treg frequencies results in stem cell/progenitor apoptosis in low-risk disease. Rather than hyperinflammation normally found in HLH patients, chronic low-grade inflammation develops associated with tumor-necrosis factor-alpha (TNF-α), interleukin 1-beta (IL-1β) and IL-6 upregulation. Cytokine abnormalities in MDS leads to increased apoptosis and marrow hypercellularity. However, decreased marrow cellularity can develop in fraction of MDS patients harboring trisomy 8 karyotypic abnormality. Interestingly, our patient and two previously published cases harbored + 8 in their metaphase analysis at MDS diagnosis while HLH had developed [3, 5]. + 8 cells induce autologous T-cell oligoclonal expansion capable to target MDS precursor/progenitors resulting in characteristic marrow hypoplasia [8, 9]. In most of MDS cases, disease propagation depends on somatic mutations acquisition allowing dysplastic transformation. Mutations in perforin-dependent cytotoxicity are classically described in primary HLH. In our case, it is conceivable that myeloid mutations induced apoptotic and differentiation defects, and “facilitated” acquisition of HLH-like phenotype.

The preferred HLH treatment is HLH-94 protocol, but its administration is limited by MDS-induced cytopenias, as was the case with our patient. This is largely due to hemopoietic progenitor depletion initiated MDS and likely aggravated by HLH. Daitoku *et al.* reports superior outcome in a HLH-MDS patient treated with Methylprednisolone, CSA and decitabine[4]. Indeed, *in vivo* administration of hypomethylating agents (HMA) induce Foxp3+ Tregs expansion leading to immune suppression, and attenuates graft-versus-host disease [10]. The combination of HMA and immunosuppressive therapy may be promising treatment in HLH-MDS cases as suggested by the interesting outcomes in the case described by Daitoku *et al*.

We acknowledge limitations to our interpretation. It is possible that hyperinflammation initiated by HLH led to marrow dysplasia. However, the evidence of +8 strongly suggests a clonal etiology supporting MDS induced HLH. Secondly, sequencing did not include HLH like mutations such as *PRF1, STX11, UNC13D, STXBP2, RAB27A, SH2D1A, BIRC4, LYST, ITK, SLC7A7, XMEN*, *HPS*, among others. This may affect our ability to assign HLH as culprit for hyperinflammation in our case. However, Rui *et al*. recently demonstrated that epigenetic perturbations induced by *DNMT3A* mutations results in aberrant stem cell gene-expression associated with immune deregulation, which may have contributed to inflammatory manifestations in our case [11]. Additionally, recent data demonstrated that myeloid specific mutations including *TET2, ASXL1*, and* DNMT3A* can induce inflammasome activation in myelodysplasia and exacerbate inflammation [12-15]. In summary, HLH associated with MDS is an aggressive entity and should prompt careful evaluation hyperinflammatory signs. It is possible that targeting MDS hematopoiesis with hypomethylating agents in combination with immunosuppressive therapy to minimize hyperinflammation could improve life-threatening HLH in MDS patients.

## Data Availability

The datasets used and/or analysed during the current study are available from the corresponding author on reasonable request.

## Abbreviations

HLH: Hemophagocytic lymphohistiocytosis
MDS: Myelodysplastic syndrome
HMA: Hypomethylating agent
